# Breast Tumor Blood Flow, Disease Progression, and Treatment Response: The Role of Exercise

**DOI:** 10.1007/s40279-026-02392-w

**Published:** 2026-01-27

**Authors:** Olívia M. Ruberti, Guilherme D. Telles, Sophie F. M. Derchain, Rafaela B. Araújo, Dhyana Lima, Henrique Bortolozo, Luís Otávio Zanatta Sarian, Eva Zopf, Rodrigo Menezes Jales, Miguel S. Conceição

**Affiliations:** 1 Health Science Program, Sao Francisco University, Avenida São Francisco de Assis, 218. Cidade Universitária, Bragança Paulista, São Paulo, 12916-900 Brazil; 2 Center of Studies in Exercise Oncology (CEEO), Campinas, Brazil; 3 Laboratory of Aging Biology (LaBE), Biology Institute-UNICAMP, Campinas, Brazil; 4 Department of Obstetrics and Gynecology, Faculty of Medical Sciences, University of Campinas, Campinas, Brazil; 5 Mary MacKillop Institute for Health Research, Australian Catholic University, Melbourne, Australia

## Abstract

Breast cancer remains one of the leading causes of cancer-related mortality in women worldwide. A key driver of tumor progression and resistance to therapy is abnormal vascularization, which leads to heterogeneous blood flow, hypoxia, impaired drug delivery, and increased malignancy. Emerging clinical evidence suggests that breast tumor Doppler ultrasound parameters can predict pathological response to neoadjuvant chemotherapy, highlighting the potential of tumor blood flow as a prognostic biomarker. In this context, strategies to modulate tumor perfusion—particularly through non-pharmacological approaches—are of growing interest. Exercise has shown promise in promoting vascular remodeling, improving oxygenation, and enhancing treatment efficacy. This narrative review discusses current evidence on the role of tumor blood flow in disease progression and therapeutic response, with special emphasis on the modulatory effects of exercise training. We also explore how tumor blood flow is assessed, briefly outlining key methodologies such as Doppler ultrasound, contrast-enhanced imaging, and perfusion-related biomarkers, and their relevance for interpreting exercise-induced vascular adaptations, its association with tumor aggressiveness, and the interplay between exercise and oncological therapies. Understanding how exercise influences tumor hemodynamics may pave the way for innovative adjuvant strategies in breast cancer management.

## Key Points

**Table:** 

Poor blood flow in breast tumors can make treatments such as chemotherapy less effective. New imaging techniques help doctors see how well blood moves through tumors and predict how patients will respond to therapy.
Exercise might improve blood flow in tumors. Animal studies show that exercise training can make tumor vessels more organized, helping oxygen and treatments reach the cancer more effectively. Most of the current evidence on exercise-induced improvements in tumor vascular function comes from aerobic training.
Using exercise as part of cancer care could enhance treatment results. Although more human studies are needed, early evidence suggests that combining exercise with standard therapies might slow cancer growth and improve patient outcomes.

## Introduction

Noncommunicable diseases are the leading cause of mortality worldwide, with cancer accounting for approximately 9.7 million deaths in 2022. Breast cancer is the most prevalent in women, responsible for approximately 6.9% of these deaths [1]. Despite advances in early detection and treatment, a significant proportion of deaths can be attributed to the biological complexity of tumors, which limits the efficacy of current therapeutic strategies and allows tumor progression. Among the various factors influencing tumor progression and treatment response, tumor vascularization plays a crucial role [2–13]. Consequently, factors affecting tumor vascularization have been under growing scrutiny in attempt to develop strategies aimed at reducing breast cancer mortality. In solid tumors, the vasculature is responsible for supplying oxygen, nutrients, and therapeutic agents to malignant tissue, and its structure and function directly influence tumor growth, metastatic potential, and treatment susceptibility [14–16].

An important characteristic of tumors is that their vascular system differs from other tissues, characterized by dysfunctional blood vessels (i.e., characterized by irregular, tortuous, and hyperpermeable vessels; poor pericyte coverage; and disorganized endothelial cell arrangement) [17]. The structure of these vessels induces heterogeneous tumor blood flow, resulting in the uneven distribution of oxygen, immune cells, and chemotherapeutic agents within the tumor during treatment [17]. Indeed, evidence shows that solid tumors exhibit both diffusive and perfusive limitations to oxygen delivery, leading to a hypoxic environment, a critical factor in treatment resistance and a more aggressive tumor phenotype [18, 19].

Given the critical role of vascular dysfunction in tumor progression, accurate assessment of the tumor blood flow is essential. Advances in imaging technologies, such as Color Doppler ultrasound, now allow for noninvasive evaluation of blood flow dynamics [20]. With Doppler ultrasound, it is possible to assess real-time blood flow dynamics by detecting frequency shifts caused by moving red blood cells [21]. In addition, contrast-enhanced ultrasound (CEUS) improves the visualization of microvascular networks by using microbubble contrast agents that enhance the detection of perfusion patterns [22]. Through these capabilities, ultrasound imaging can help monitor vascular remodeling induced by therapeutic interventions, assess tumor perfusion heterogeneity, and predict clinical outcomes, such as complete pathological response (pCR), ultimately serving as a valuable tool for evaluating treatment efficacy and disease prognosis [23]. Clinical evidence has already suggested a relationship between blood flow parameters measured by Color Doppler and treatment efficacy (i.e., the response to neoadjuvant chemotherapy) [24]. For instance, Conz et al*.* demonstrate that women with non-luminal tumors and an end-diastolic velocity (EDV) above 1.9 cm/s had a 35% higher chance of achieving pCR than those with lower values after two neoadjuvant chemotherapy (NACT) cycles, suggesting a clinically relevant link between tumor blood flow and treatment response [23]. This finding suggests that elevated end-diastolic blood flow may be indicative of more perfused or vascularized tumors, potentially facilitating better drug delivery and response to treatment.

While imaging techniques provide valuable insights into tumor perfusion, strategies to actively modulate blood flow remain underexplored in clinical oncology. Considering evidence showing that increased blood tumor flow can improve clinical responses in the cancer context, strategies to improve tumor vascularization and blood flow may play a promising role in cancer treatment. In this context, preclinical studies suggest that exercise can enhance intratumoral vascularization [18, 25, 26]. For example, in a preclinical study, long-term voluntary wheel running has been shown to improve blood perfusion and vascularization in an animal model orthotopically implanted with human breast cancer cells, likely through exercise-induced upregulation of angiogenic factors such as vascular endothelial growth factor (VEGF) and enhanced endothelial function [26]. However, the mechanisms and translational potential still require further investigation. Despite emerging evidence, the clinical literature remains fragmented, with limited integration of mechanistic insights, heterogeneous methodologies, and a lack of consensus regarding the magnitude and clinical relevance of exercise-induced vascular modulation in human tumors. These gaps highlight the need for comprehensive analyses capable of synthesizing current knowledge and identifying priority areas for future research. This narrative review examines the regulation of tumor blood flow, its relationship with oxygenation and malignancy, and the effects of oncological therapies. Furthermore, we explore the potential of exercise to modulate tumor perfusion, its underlying mechanisms, and implications for treatment response*.*

## Tumor Characteristics, Pathological Angiogenesis and Clinical Implications

### Hallmarks of Cancer: Role of Angiogenesis and Tumor Vascular Abnormalities

Initially proposed by Robert A. Weinberg and Douglas Hanahan, the Hallmarks of Cancer describe essential functional capabilities that human cells acquire during their progression from normal to malignant states, providing a framework to understand common cellular features across various cancer types [27, 28]. These include sustaining proliferative signaling, evading growth suppressors, resisting cell death, enabling replicative immortality, inducing/accessing vasculature (pathological angiogenesis), activating invasion and metastasis, reprogramming cellular metabolism, and avoiding immune destruction. This framework helps to rationalize the complex biology of tumors and their diverse pathways of progression [29, 30].

Recent evidence shows that tumor vascularization is particularly critical to tumor progression, as it regulates oxygen and nutrients to support uncontrolled cell proliferation while also serving as a gateway for metastatic dissemination. The ability of tumors to induce and manipulate blood vessel formation not only sustains their growth but also plays a key role in influencing their microenvironment and response to cancer therapy [20, 29]. As a result, tumor tissue acquires distinct structural and functional features that differ markedly from normal vasculature.

**Fig. 1:**
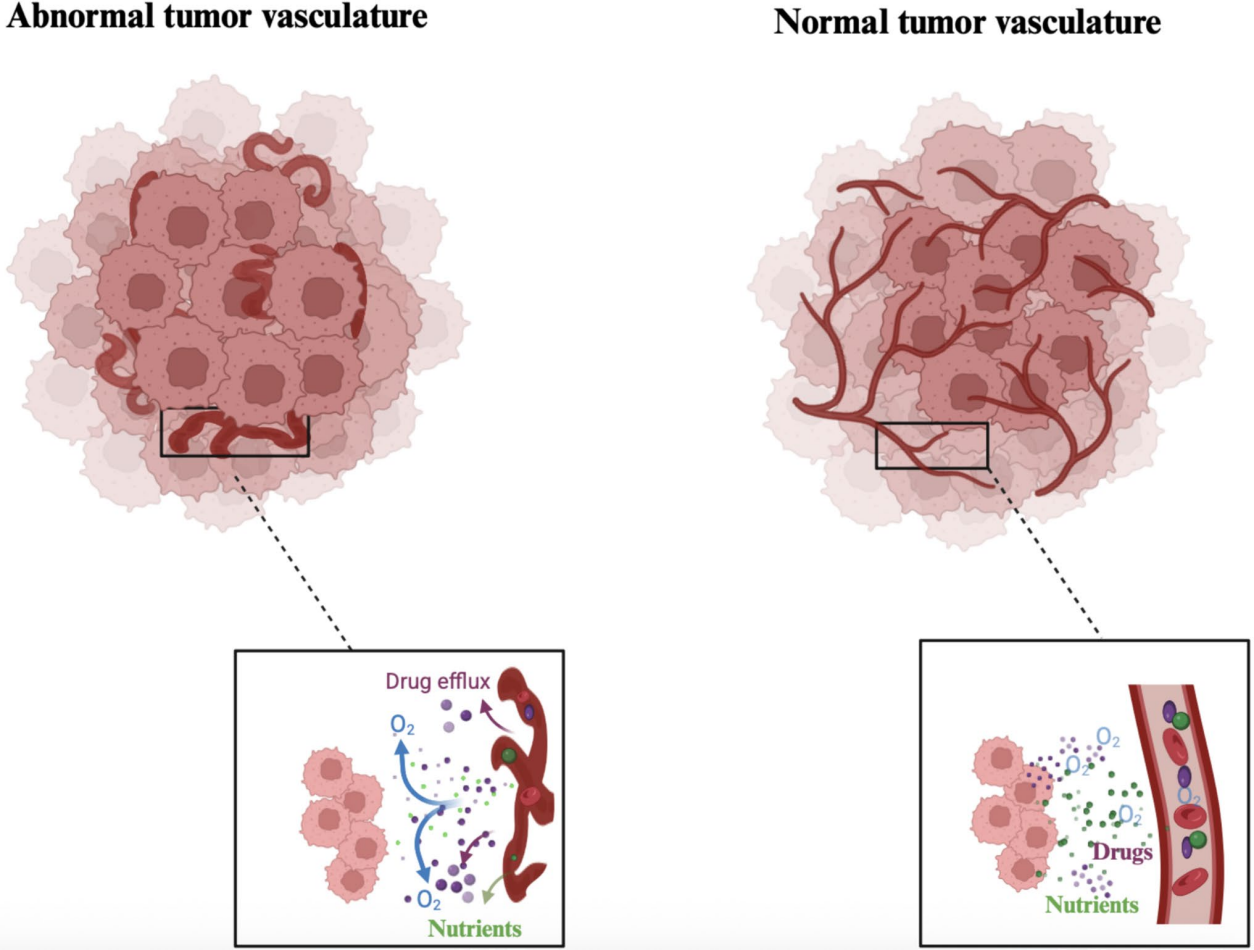
Tumor vascular normalization improves oxygen and drug delivery. Schematic representation of abnormal (left) and normalized (right) tumor vasculature. Abnormal tumor vessels are disorganized, tortuous, and leaky, leading to hypoxia and impaired drug delivery owing to inefficient oxygen diffusion and increased drug efflux. In contrast, normalized vasculature exhibits more organized and functional vessel architecture, enabling enhanced perfusion, improved oxygenation, and better delivery of therapeutic agents and nutrients to the tumor microenvironment. Created with BioRender (Licence: KE292C3JJO https://BioRender.com/406rdhr)

During organogenesis (i.e., the process of organ formation and development during embryogenesis), the close anatomical and functional association between blood vessels and the developing parenchyma is maintained by their coordinated growth. However, in adult tissues, angiogenesis is a transient and tightly regulated process. Conversely, tumors are characterized by an abnormal vascular system and heterogeneous blood flow, and this vascularization may be correlated with the degree of tumor malignancy [20]. Proliferating cells in early lesions initially lack angiogenic capacity, limiting their expansion. Incipient neoplasms must acquire this ability to grow further [27]. During tumor progression, an “angiogenic switch” is activated and remains on, leading to continuous blood vessel growth that supports tumor expansion. This process is regulated by opposing factors that either promote or inhibit angiogenesis. These factors include signaling proteins that bind to receptors on endothelial cells. For instance, vascular endothelial growth factor A (VEGF-A) is a key pro-angiogenic factor that stimulates endothelial cell proliferation, migration, and survival, primarily through activation of the VEGF receptor-2 (VEGFR-2) pathway. Conversely, thrombospondin-1 (TSP-1) is a matricellular glycoprotein that inhibits angiogenesis by interacting with CD36 on endothelial cells, leading to apoptosis and suppression of vascular growth. The balance between these factors determines the extent of tumor vascularization [27, 29]. In addition, integrin signaling is crucial, as quiescent vessels and sprouting capillaries express different integrin classes. Disrupting this signaling can inhibit angiogenesis, highlighting the importance of cell adhesion and extracellular proteases in regulating this process [27]. This dysregulated and sustained angiogenic signaling gives rise to structurally and functionally abnormal vasculature. Compared with the well-organized and hierarchically structured vessels of healthy tissues, tumor blood vessels display excessive branching, irregular diameters, uneven spatial distribution, and increased permeability (Fig. 1) [20, 31]. These abnormalities are driven by the overexpression of pro-angiogenic factors, such as VEGF, within the tumor microenvironment, resulting in a chaotic vascular network that is often fragile, leaky, and poorly perfused. This aberrant vascular architecture compromises efficient oxygen and drug delivery, contributes to hypoxia and metabolic stress, and plays a key role in therapy resistance, underscoring the clinical importance of targeting tumor vasculature.

### Hypoxia and Metabolic Consequences

The supply of nutrients and oxygen by the vascular system is crucial for cell function and survival. However, the poor structural integrity of tumor blood vessels is often accompanied by a lack of proper pericyte coverage (i.e., contractile cells associated with capillary and venule walls that regulate blood flow, maintain the blood–brain barrier, control vascular permeability, and support angiogenesis) and abnormal basement membrane composition, which contribute to vessel immaturity and dysfunction [31]. As a result, tumor vascularization is inefficient, creating regions of severe nutrient deprivation and hypoxia that further drive tumor progression, metabolic reprogramming, and resistance to therapy, enhancing metastatic potential [32–34]. Nutrient deprivation can alter translational control, intensifying the malignant phenotype of breast cancer cells, an effect that may be directly linked to inadequate vascularization and the limited availability of essential blood-borne nutrients [27]. In the poorly supplied regions, cellular metabolism is profoundly affected: the lack of oxygen (hypoxia) forces cells to ferment glucose, leading to lactate accumulation and a reduction in local pH [35]. Simultaneously, the scarcity of essential nutrients (such as glucose and amino acids), along with the accumulation of metabolic waste products (e.g., lactic acid and free radicals) intensifies cellular stress. As these conditions impose strong selective pressure, many cells fail to survive, but those that develop metabolic and molecular adaptations can persist [35]. Studies in noninvasive breast cancer (ductal carcinoma in situ) have shown that poorly vascularized central areas undergo evolutionary selection of adapted cells, and over time, only cells capable of adjusting their metabolism to resource scarcity survive, emerging with more aggressive phenotypes [35, 36]. In summary, chronic nutrient and oxygen deprivation within the tumor microenvironment favors metabolically resilient cellular variants, enhancing their ability to survive, proliferate, and disseminate [35].

Since many chemotherapeutic agents rely on forming reactive oxygen species to induce apoptosis in cancer cells, tumor hypoxia (i.e., suboptimal oxygen levels in the tissues of living organisms) reduces the efficacy of these drugs [17]. Furthermore, hypoxia activates hypoxia-inducible factor 1 (HIF-1), which in turn, induces the transcription of genes involved in crucial aspects of tumor biology, such as angiogenesis, cell survival, invasion, and glucose metabolism [37]. HIF-1-induced angiogenesis in solid tumors paradoxically results in pathological angiogenesis, characterized by abnormal blood vessel formation, impaired perfusion, and consequently, poor tumor oxygenation [38]. This, in turn, perpetuates the cycle, driving further tumor proliferation. In this context, the abnormal vascular system observed in breast tumors, characterized by low tumor blood flow and dysfunctional angiogenesis, can promote nutrient deprivation, metabolic reprogramming, hypoxia and tumor progression, negatively impact drug delivery (e.g., chemotherapy), and increase the likelihood of metastasis [18, 34].

### Clinical Implications

Studies on endometrial carcinomas indicate that low tumor blood flow, combined with increased capillary leakage and high microvascular proliferation, is associated with reduced recurrence/progression-free survival [17, 39]. This association is likely due to the synthesis of blood vessels (i.e., tumor angiogenesis), which, despite being abundant, are heterogeneous, irregular, and exhibit rough vascular lumens, rendering them abnormal in almost every aspect of their structure and function [31]. They exhibit excessive branching, distorted structure, micro hemorrhaging, and increased permeability. In addition, endothelial cells show abnormal proliferation and apoptosis levels [29, 40]. Such characteristics result in irregular tumor blood flow, leading to an inconsistent distribution of oxygen, nutrients, immune cells, and drugs such as chemotherapy. Together, these factors could impact the treatment response [17].

In addition to presenting an abnormal vascular system, tumor angiogenesis may be associated with significant metabolic changes in the tumor that could influence disease progression and treatment resistance. Metabolomic studies in endometrial cancer suggest that lipid species (e.g., lysophospholipids) and resolvin D correlate with tumor blood flow and survival [16, 40], highlighting potential biomarkers for vascular function in breast cancer [17, 41].

In summary, tumor vasculature is structurally and functionally aberrant, perpetuating hypoxia, metabolic stress, and treatment resistance. This self-reinforcing cycle underscores the need for strategies to modulate vascular function—whether through anti-angiogenic therapies, vascular normalization, or non-pharmacological interventions. Accurate assessment of tumor blood flow is essential to support the implementation, development of such interventions, and monitor their effects.

## Assessing Tumor Blood Flow

Imaging techniques play a pivotal role in the diagnosis, risk stratification, staging, prognosis, and individualized treatment planning of breast cancer. Diagnostic imaging not only detects and characterizes lesions but also classifies them into malignancy risk categories, guides image-assisted biopsy procedures, complements physical examination in locoregional staging, monitors response to neoadjuvant therapy, and assists in tailoring surgical planning for each patient. Therefore, the methodologies discussed below were selected on the basis of their clinical applicability, reproducibility, availability in oncologic practice, and growing validation as indicators of vascular function rather than solely anatomical structure.

Ultrasonography is indicated for evaluating breast lesions in women of all ages, particularly owing to its ability to differentiate between fibroglandular parenchyma and benign and malignant tumors, which enhances its sensitivity in dense breasts. It improves the assessment of breast masses and has evolved beyond simple morphological assessment [20, 23]. In addition to evaluating lesion size, echotexture, and morphology, ultrasound now enables quantitative evaluation of tumor stiffness through shear wave elastography and real-time assessment of tumor perfusion using color and spectral Doppler imaging [20, 23]. These Doppler techniques estimate blood flow velocity and direction by detecting frequency shifts caused by moving erythrocytes, offering indirect yet valuable insight into the tumor microcirculation. Notably, ultrasonography is noninvasive, widely accessible, contrast-free, and comparatively safer than magnetic resonance imaging, particularly in settings with limited resources [20, 23].

Recent advances in ultrasonographic imaging—such as high-definition flow (HDF) imaging, 3D power Doppler, and improvements in transducer technology—have significantly enhanced the sensitivity of vascular signal detection and the visualization of fine vascular structures within breast tumors [20]. These innovations allow for detailed assessment of vascular morphology, including vessel tortuosity, branching pattern, and caliber irregularity, as well as flow characteristics. Such features are critical for differentiating benign from malignant lesions, with malignant tumors typically exhibiting tortuous, spiculated, or radial vessels and chaotic branching, while benign lesions often display straighter or peripheral vascular patterns [42]. In addition, Doppler velocimetry parameters—such as peak systolic velocity (PSV), EDV, resistive index (RI), and pulsatility index (PI)—are increasingly used as noninvasive indicators of tumor vascular resistance and perfusion dynamics [24]. These Doppler-based indices reflect distinct aspects of vascular physiology: PSV indicates peak flow during systole, EDV reflects residual flow in diastole, RI provides a measure of downstream vascular resistance, and PI captures pulsatility. Together, they offer clinically relevant insights into intratumoral hemodynamics, which may change during treatment and correlate with therapeutic response. Quantitative parameters, such as vessel number, blood flow volume, and flow velocity, may change in response to NACT, potentially reflecting vascular remodeling or treatment efficacy. As such, their incorporation into clinical protocols is under active investigation [24, 42]. Given that exercise has been shown to influence vascular tone, shear stress, angiogenic signaling, and perfusion regulation, these imaging-derived indices constitute meaningful surrogate markers for evaluating physiological pathways potentially modulated by exercise training.

Several recent studies underscore the diagnostic and biological relevance of these techniques. Niu et al. demonstrated that increased maximum velocity (*V*_max_) and decreased RI on Doppler imaging were significantly associated with higher expression of angiogenesis and proliferation markers, including vascular endothelial growth factor 165 (VEGF165), neuropilin-1 (NRP-1), cluster of differentiation 31 (CD31), and Yes-associated protein (YAP), as well as lower levels of key tumor-suppressor proteins such as phosphatase and tensin homolog (PTEN) and mitofusin-2 (MFN2) in patients with breast cancer [43]. Dong et al. found that contrast-enhanced ultrasound with micro-flow imaging (CEUS-MFI) had greater sensitivity (94.4%) and diagnostic accuracy (91.5%) compared with CEUS or conventional Doppler, particularly in characterizing microvascular morphology and distribution [44]. Mohindra et al. reported that non-contrast microvascular techniques such as Angio-PLUS, which detect low-flow signals and microvessels more effectively than conventional Doppler, yielded higher vascular scores in malignant lesions and significantly improved diagnostic accuracy [45]. Angio-PLUS vascular pattern descriptors (e.g., mesh and radial types) strongly correlated with histopathology and improved the differentiation between benign and malignant tumors [45, 46]. Similarly, Luo et al. demonstrated that micro-flow imaging (MFI) and HD-MFI techniques detected more vascular signals, especially penetrating and irregular patterns (e.g., “crab claw-like”), compared with Color Doppler Flow Imaging (CDFI), and could downgrade BI-RADS 4A lesions with high safety, potentially reducing unnecessary biopsies [47]. Taken together, advanced Doppler-based techniques not only enhance the characterization of tumor vascularity but also provide functional insights that complement morphological assessment.

Recent studies have further explored how ultrasonography—including Doppler and elastography—can reflect underlying tumor biology, particularly in relation to breast cancer molecular subtypes and treatment response [23, 48]. For instance, luminal A tumors are typically associated with spiculated margins, reduced blood flow, and decreased stiffness, whereas luminal B tumors often display angular margins. HER2-positive tumors are characterized by increased blood flow, calcification, and greater stiffness [48]. Moreover, Doppler ultrasound parameters can predict pathological complete response (pCR) in patients undergoing NACT. Notably, EDV measured after two NACT cycles has demonstrated the highest predictive value for pCR in non-luminal tumors [23]. Altogether, ultrasonography has been increasingly recognized as a valuable tool for improving breast cancer prognosis.

Technological improvements now allow detailed evaluation of vessel caliber and tortuosity. Malignant tumors often develop vessels with heterogeneous, dilated calibers and tortuous paths, reflecting disorganized angiogenesis [49, 50]. These imaging advancements provide higher-resolution views of vascular morphology and aid in distinguishing malignant from benign tumors, thus supporting staging, treatment planning, and therapeutic monitoring [20, 50]. According to Conz et al., morpho-functional parameters obtained through Doppler ultrasound, including EDV, PSV, MSV, PI, and RI, can assist in the early identification of non-luminal breast cancers more likely to respond to NACT. When measured after the initial cycles of NACT, these vascular parameters reflect changes in tumor perfusion and vascular resistance, which often correlate with treatment response. Importantly, automated and standardized measurements improve reproducibility and facilitate reliable comparisons across imaging centers. By providing a noninvasive, repeatable method to monitor tumor vascular dynamics, Doppler ultrasound contributes not only to diagnosis but also to prognostication. Early identification of responsive tumor subtypes can inform therapeutic decisions, including whether to continue NACT, intensify treatment, or consider early surgical intervention [23]. Supporting these findings, Zhang et al. demonstrated that changes in tumor blood supply assessed by ultrasound after two cycles of NACT were independently associated with pathological complete response (pCR), alongside clinical variables such as histological grade, HER2 status, and the pretreatment albumin-to-alkaline phosphatase ratio (AAPR). In their cohort of 176 patients, a nomogram incorporating these parameters—including vascular changes—showed strong predictive performance for pCR. These results further highlight the value of integrating vascular imaging with clinical and biochemical markers to guide individualized treatment strategies in breast cancer [51]. While identifying tumor masses for precise measurement requires trained operators, such expertise can typically be acquired within a few months in specialized centers [23].

In summary, Doppler ultrasound offers a practical, reproducible, and increasingly validated method for assessing tumor perfusion in breast cancer. As evidence supporting its clinical utility grows, further validation and standardization of acquisition protocols will be essential for its broader integration into routine oncology practice.

## Effects of Exercise on Tumor Blood Flow

Hypoxia and inadequate blood supply contribute to tumor aggressiveness and reduce the effectiveness of systemic therapies (e.g., chemotherapy). Normalizing tumor vasculature, in turn, can enhance oxygenation, facilitate drug delivery, and improve chemotherapy response. Indeed, the substantial body of evidence supporting the therapeutic benefits of tumor vascular normalization now presents a compelling opportunity to enhance the efficacy and outcomes of cancer treatments [33, 52]. Exercise may play an important role in tumor vascular normalization by increasing physiological perfusion and vascularization, as observed in preclinical and clinical solid tumor models [42, 53–55]. For instance, a study demonstrated that exercise may enhance radiotherapy effectiveness in prostate cancer by improving tumor perfusion, reducing hypoxia, and increasing natural killer cell infiltration, although these radiosensitising effects and the optimal exercise prescription still need confirmation in preclinical and clinical trials [42]. Thus, exercise emerges as a physiologically driven, non-pharmacologic strategy to improve vascular integrity in tumors [52].

In addition to its vascular effects, improved tumor blood flow may facilitate the trafficking of immune effector cells into the tumor microenvironment, representing another key mechanism through which exercise can influence tumor biology. A study by Pedersen et al. demonstrated that endurance exercise reduces tumor growth in mouse models of cancer through increased infiltration and activation of natural killer (NK) cells [56]. Also, several preclinical studies have reinforced this concept, showing that exercise enhances immune cell recruitment, cytotoxic activity, and overall anti-tumor immunity across different solid tumors [57–59]. Collectively, these findings suggest that exercise-induced increases in tumor perfusion may not only improve oxygenation and treatment delivery but also support immune cell access to tumor tissue, thereby creating a more immunologically permissive microenvironment conducive to tumor control. Although most evidence comes from animal models, the underlying principles likely extend to a broad range of solid tumors and underscore the need for clinical studies integrating vascular and immune outcomes in exercise oncology research.

Aerobic training appears to have pro-vascular and angiogenic effects (i.e., physiological angiogenesis) in patients with various comorbidities [18]. Specifically, in cancer, studies suggest that aerobic training can improve the perfusion of solid tumors and mitigate tumor hypoxia [53, 54]. This effect is mediated by increased shear stress (i.e., mechanical forces generated by blood flow), which regulates endothelial cell function and vascular remodeling. Moreover, exercise training seems to promote a more homogeneous distribution of blood flow within tumors and increase baseline oxygenation, indicating a persistent modulation of the tumor microenvironment [19]. In addition, aerobic exercise has been shown to exert an anti-Warburg effect, enhancing lactate clearance capacity. As lactate plays a key role in carcinogenesis and is linked to tumor progression, including cell migration, angiogenesis, immune escape, and metastasis, its reduction may help suppress tumor proliferation and progression [60].

Building on these adaptations, it is also essential to consider the immediate physiological responses triggered by individual exercise bouts, which initiate many of the vascular mechanisms later consolidated through training. Acute bouts of exercise increase cardiac output and regional blood flow, generating elevated shear stress along vessel walls. This mechanical stimulus activates endothelial mechanosensors, leading to phosphorylation of endothelial nitric oxide synthase (eNOS) and increased nitric oxide (NO) production, which drives vasodilation and transient increases in perfusion [80–82]. Repeated exposure to shear stress promotes endothelial alignment, enhances barrier integrity, and shifts vascular signaling toward a more quiescent, normalized phenotype [80, 81]. Exercise also stimulates the release of multiple myokines, such as VEGF, IL-6, irisin, and angiopoietin-1, which act systemically to promote angiogenesis, endothelial activation, and vascular remodeling. These factors support pericyte recruitment, vessel maturation, extracellular matrix reorganization, and improved perfusion efficiency [83, 84]. Together, these shear-stress and myokine-mediated pathways counteract the structurally abnormal, tortuous, and hypoxic vasculature characteristic of malignant tumors, creating a microenvironment more permissive to drug delivery and immune infiltration.

The acute and cumulative mechanistic effects translate into measurable structural and functional changes in the tumor vasculature, as demonstrated by recent preclinical evidence. Recent work demonstrates that aerobic exercise training induces tumor vascular normalization in preclinical models [52]. Indeed, studies have shown that exercise significantly enhances intratumoral perfusion and vascularization in orthotopic breast and prostate cancer models [18, 25, 26]. Another study showed that immunocompetent female mice with orthotopically implanted syngeneic 4T1 murine breast cancer cells in the dorsal mammary fat pad exhibited reduced tumor growth following 18 days voluntary wheel running (i.e., 24-h ad libitum wheel running). This reduction was associated with increased apoptosis, microvessel density, vascular maturity and perfusion, and decreased intratumoral hypoxia. In the same study, the combination of exercise and chemotherapy (cyclophosphamide) further reduced tumor growth compared with chemotherapy alone [32]. These findings suggest that aerobic training shifts the tumor microenvironment toward a state of less heterogeneous vascularization, improved perfusion, and reduced hypoxia. According to the authors, since hypoxia is a major barrier to the efficacy of chemotherapy, combining aerobic exercise with chemotherapy could be an effective strategy to enhance chemosensitivity in solid tumors [18]. Thus, understanding how exercise bouts or training affects tumor physiology is critical.

Several mechanisms through which exercise training may impact vascularization in the tumor microenvironment have been proposed. Exercise increases shear stress, which elevates angiogenic factors such as VEGF-A, osteopontin, and macrophage inflammatory protein-1α (MIP1α) within endothelial cells. This process activates the transcriptional factor nuclear factor of activated T cells (NFAT), thereby stimulating angiogenesis. In addition, it is well-established that exercise training enhances the bioavailability of nitric oxide, a critical vasodilator and mediator of angiogenesis, vascular maturity, and lymphatic vessel function [16, 38]. Exercise bouts sessions can also directly influence tumor vascular physiology [38, 61]. For example, a preclinical study using a prostate cancer model revealed that a single session of aerobic exercise increased tumor blood flow by approximately 200% as well as the number of patent vessels. Tumor hypoxia was reduced by 50% during the exercise session, and maximal norepinephrine-induced vasoconstriction in tumor arterioles was attenuated by about 95% compared with control prostate vessels [54]. Recent reviews have further emphasized the capacity of exercise to modulate the tumor microenvironment, including enhancements in immune cell infiltration, angiogenic regulation, and vascular function, thereby reinforcing the biological rationale for exercise as a therapeutic adjuvant in cancer care [38].

Although aerobic exercise has traditionally received the most attention in the context of tumor vascular modulation, it is important to emphasize that evidence regarding resistance exercise remains limited. Only a small number of preclinical studies have explored how muscle contraction per se may influence tumor biology and vascular function. Nonetheless, contraction-derived myokines released during resistance exercise, including IL-6, IL-15, and irisin, have been shown to modulate angiogenesis, suppress tumor cell proliferation, and enhance immune surveillance [62–64]. Also, Hojman et al. identified oncostatin M (OSM) in exercise-conditioned serum and muscle-derived media as a key myokine mediating the inhibitory effects of exercise on mammary cancer cell growth [62]. Despite these emerging mechanistic insights, no clinical trials have isolated the direct effects of resistance exercise on tumor vascularization in humans. This represents an important gap, as combining aerobic and resistance modalities may amplify complementary physiological stimuli capable of normalizing tumor vasculature and enhancing therapeutic responsiveness.

**Fig. 2:**
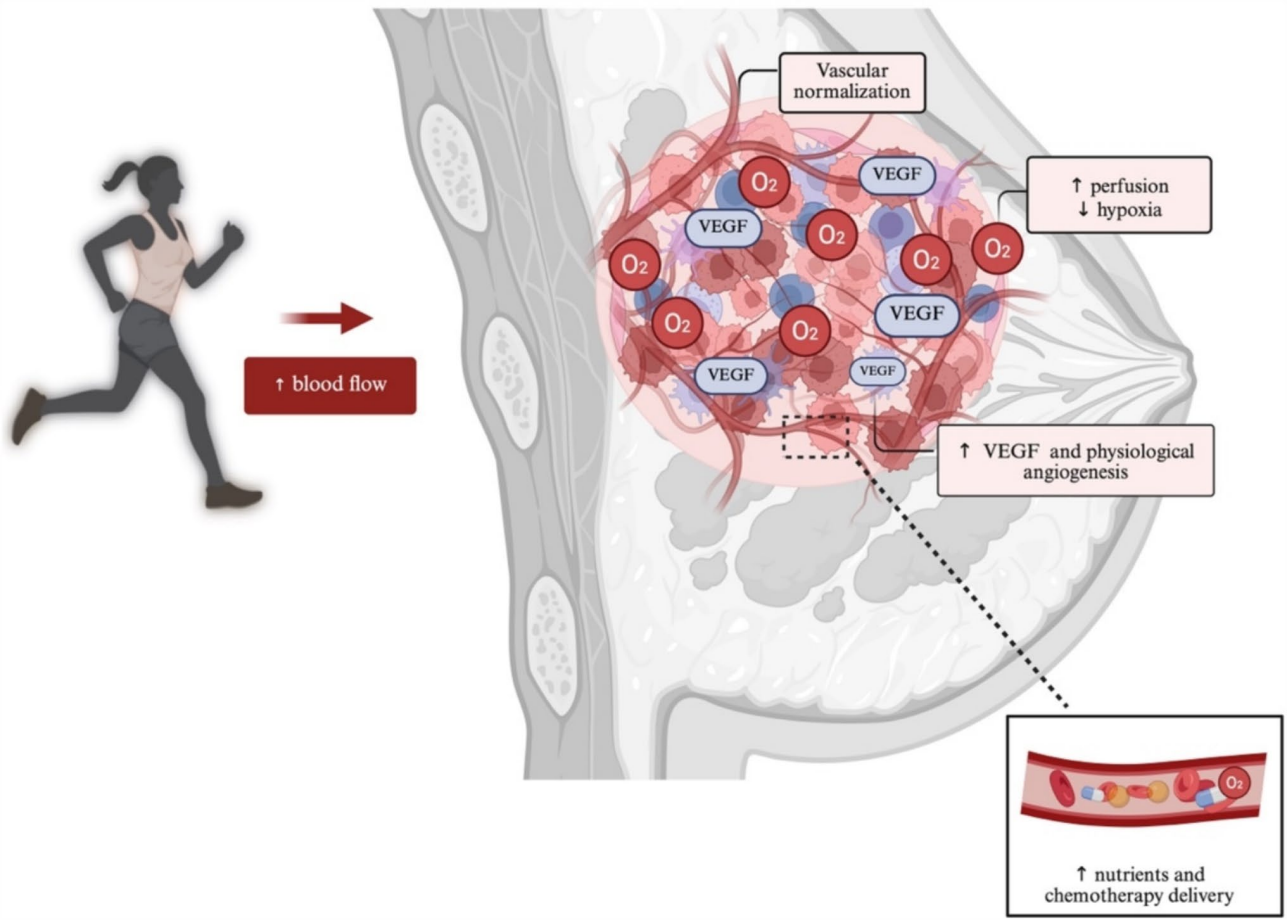
Effects of exercise on tumor vascularization. Exercise training increases blood flow and VEGF expression, leading to vascular normalization and physiological angiogenesis. These effects contribute to enhanced tumor perfusion, reduced hypoxia, and improved delivery of nutrients and chemotherapeutic agents, potentially enhancing the response to cancer treatment. VEGF, vascular endothelial growth factor. Created with BioRender (Licence: NA292C38AS https://BioRender.com/73wns0i)

While preclinical studies have demonstrated the influence of exercise training on blood flow and its subsequent effects on tumors, clinical studies evaluating its effects on blood flow and tumor growth in oncologic patients remain scarce. To date, only one study by Jones et al. has attempted to investigate the effects of aerobic training combined with doxorubicin–cyclophosphamide NACT compared with chemotherapy alone on breast tumor blood flow. However, this study was unable to accurately assess the impact of exercise on blood flow owing to its small sample size (five patients in the training + chemotherapy group and two patients in the chemotherapy group), being considered only a pilot study [18]. While more human studies are needed, our hypothesis is that exercise training also has the potential to increase tumor blood flow in oncologic patients, creating a microenvironment that is less favorable for cancer progression and more responsive to therapy (Fig. 2). Improved vascular function could facilitate better oxygenation and enhance the delivery of chemotherapeutic agents, thereby increasing treatment efficacy. In addition, exercise-induced changes in blood flow may alter signaling pathways involved in angiogenesis, leading to a more normalized vascular network. This effect is particularly relevant, as tumor vascular abnormalities are associated with increased metastatic potential and treatment resistance [65]. If exercise can promote a more functional vasculature, it may serve as an effective adjuvant therapy to enhance chemotherapy outcomes and potentially reduce disease recurrence.

Despite its promising potential, exercise oncology remains a field of research that requires further investigation, particularly regarding tumor vascularization. The lack of controlled clinical studies investigating the direct effects of exercise bouts or training on tumor blood flow highlights a critical gap in literature. Future research should establish standardized protocols for assessing tumor hemodynamics in response to exercise interventions, ensuring reproducibility and clinical relevance. Understanding the mechanisms by which exercise influences tumor vasculature could shed light on novel therapeutic strategies that integrate exercise as a complementary approach to conventional cancer treatments. Further investigations in this area could have significant implications for improving patient outcomes and personalizing treatment strategies.

## Clinical Applications and Future Directions

### Clinical Implications for Exercise Prescription

Building on the preclinical and mechanistic evidence previously discussed, there is increasing clinical interest in how exercise might be incorporated as an adjuvant strategy to modulate tumor vascularization and improve treatment outcomes in cancer care. While data on these outcomes in humans are still emerging, existing evidence is encouraging and provides a rationale for integrating exercise into oncology practice [18]. Notably, a randomized controlled trial by Sanft et al. investigated the effects of a lifestyle intervention combining exercise and nutrition in women with breast cancer undergoing chemotherapy. Although the intervention did not significantly alter chemotherapy relative dose intensity (RDI), a subgroup analysis of patients receiving neo-adjuvant chemotherapy (*n* = 87) revealed that patients in the intervention group exhibited a significantly higher rate of pathologic complete response (pCR) compared with usual care (53 versus 28%), particularly among those with hormone receptor-positive/HER2-negative and triple-negative tumors [66]. Another study, the BENEFIT trial, also examined the effects of supervised aerobic or resistance training during neoadjuvant chemotherapy in patients with breast cancer. Exercise promoted greater tumor shrinkage and pCR in hormone receptor-positive tumors and helped preserve relative dose intensity in hormone-receptor-negative cases [67]. These findings underscore the potential of non-pharmacological strategies to enhance tumor sensitivity to neoadjuvant therapy, supporting the hypothesis that exercise may influence tumor biology beyond systemic health benefits.

To better contextualize these clinical findings, it is important to distinguish between the acute and chronic physiological responses to exercise, as each may contribute differently to tumor vascular modulation. Acute exercise bouts elicits immediate hemodynamic effects, including transient increases in cardiac output, shear-stress-dependent endothelial stimulation, and rapid nitric oxide (NO)-mediated vasodilation [68, 69]. These responses can temporarily enhance perfusion and oxygenation, potentially creating therapeutic windows that could improve chemotherapeutic drug delivery when exercise is synchronized with treatment [26]. In contrast, long-term exercise (i.e., exercise training) induces more durable structural and molecular adaptations, such as increased endothelial stability, enhanced pericyte coverage, long-term upregulation of eNOS, and improved angiogenic and arteriogenic remodeling [70–72]. Repeated exposure to shear stress drives vascular normalization and improves perfusion efficiency [68, 73]. Distinguishing these acute versus chronic effects helps contextualize how exercise may potentiate treatment efficacy and underscores the importance of timing, frequency, and duration when integrating exercise into oncology protocols.

From a practical standpoint, moderate-intensity aerobic training (e.g., 60–75% of heart rate reserve), performed 3–5 times per week, has been associated with safety, tolerability, and broad physiological benefits in cancer populations [74, 75]. Although such prescriptions are not yet specifically tailored to modulate tumor perfusion, the mechanistic overlap with cardiovascular training responses (e.g., increased shear stress, nitric oxide bioavailability) reinforces their potential vascular effects. Moreover, recommendations from the American College of Sports Medicine (ACSM) and the Exercise Is Medicine in Oncology initiative emphasize individualized exercise prescription, taking into account cancer type, treatment phase, comorbidities, and physical function [75]. For tumors where perfusion may impact drug efficacy (e.g., breast, prostate, endometrial), integrating aerobic exercise into treatment plans, particularly before or alongside chemotherapy, may offer additional therapeutic benefits. However, clinical translation requires caution. Tumor vascular responses may vary substantially by subtype, as HER2-positive and triple-negative breast cancers typically exhibit higher microvessel density, greater angiogenic signaling (e.g., VEGF overexpression), and increased perfusion, whereas luminal A tumors tend to display lower vascularity, reduced Doppler flow indices, and a more quiescent angiogenic profile [43, 76, 77]. Individual factors, including baseline fitness, tumor biology, and systemic inflammatory status, can modulate both vascular and oncological outcomes.

Considering these findings, incorporating exercise into clinical protocols may not only help preserve physical function, manage treatment-related side-effects, and improve treatment adherence but also directly reduce tumor development. Although evidence in humans is still emerging, preliminary data suggest that exercise might influence tumor biology, including vascular remodeling and response to therapy. However, variability in tumor vascular responses and the lack of standardized clinical exercise protocols remain important challenges. Further investigation is needed to determine how exercise can be optimally applied alongside oncological care.

### Limitations of Current Evidence and Methodological Gaps

Despite promising findings from preclinical and pilot clinical studies, the integration of exercise as a modulator of tumor blood flow in oncology remains constrained by significant scientific and methodological limitations. One major challenge is the heterogeneity of study designs, including variability in cancer types and stages, which impairs the generalizability of results. Most available evidence derives from preclinical models**,** particularly murine studies using orthotopic breast or prostate cancer models, which, although valuable, do not fully replicate the complexity and interindividual variability of human tumor biology [25, 32].

Mechanistic studies in humans remain limited. In clinical settings, randomized controlled trials assessing exercise-induced modulation of tumor perfusion are scarce. The only available human data comes from one underpowered pilot trial [18]. In addition, tumor perfusion remains difficult to assess in a noninvasive and reproducible manner. Techniques such as Doppler ultrasound, dynamic contrast-enhanced MRI (DCE-MRI), and contrast-enhanced ultrasound (CEUS) offer valuable tools for improving our understanding of tumor blood flow and may contribute to the development of standardized acquisition protocols and reliable perfusion biomarkers in the exercise oncology setting [78].

Also, it remains unclear whether dose/intensity-response relationships exist (e.g., whether higher-intensity exercise leads to proportionally greater improvements in perfusion), or whether benefits plateau beyond a certain threshold. Another limitation is the lack of molecular stratification in existing clinical trials. Tumors differ in their angiogenic profiles, perfusion status, and metabolic adaptability, which likely affect their responsiveness to vascular modulation through exercise [17]. Without accounting for molecular subtype in the analysis, histological type, or hypoxia markers, clinical results may overlook relevant subgroups that stand to benefit the most from exercise interventions.

Although preclinical work has elucidated potential pathways [53, 54], the mechanisms involved have yet to be confirmed in human tumors following exercise intervention. Developing and validating biomarkers that reflect exercise-induced vascular changes in tumors (e.g., perfusion indices, hypoxia markers, endothelial function) is crucial to enable mechanistic insight and patient selection. Altogether, while the biological rationale and preliminary data support the role of exercise in modulating tumor perfusion, its clinical translation remains limited, primarily owing to the scarcity of human trials, alongside methodological inconsistencies, small sample sizes, and the absence of validated biological endpoints. These gaps hinder our ability to draw firm conclusions and highlight the need for well-powered, mechanistically informed studies to establish the clinical relevance of exercise-induced tumor adaptations.

### Future Research Directions

Clinical evidence has already suggested a relationship between blood flow parameters measured by Doppler and the response to NACT, indicating greater delivery of chemotherapeutic agents in tumors with high blood flow [24]. Our hypothesis is that exercise training in oncologic patients has the potential to increase tumor blood flow, creating a microenvironment that is less favorable for cancer progression and more responsive to therapy. Improved vascular function could facilitate better oxygenation and enhance the delivery of chemotherapeutic agents, thereby increasing treatment efficacy. In addition, exercise-induced changes in blood flow may alter signaling pathways involved in angiogenesis and this effect is particularly relevant, as tumor vascular abnormalities are associated with increased metastatic potential and treatment resistance [65]. Yet, findings from preclinical studies remain inconsistent, as methodological heterogeneity has limited the ability to determine whether aerobic exercise truly improves tumor hypoxia, vascularisation, or blood flow [79]. Improvements in perfusion and oxygen supply not only inhibit tumor progression directly but also synergize with treatments such as chemotherapy or radiation by overcoming one of their major limitations, the heterogeneous drug delivery and hypoxia in tumors. If exercise can promote a more functional vasculature, it may serve as an effective adjuvant therapy to enhance chemotherapy outcomes and potentially reduce disease recurrence.

The molecular mechanisms underlying exercise-induced vascular modulation remain under investigation. Chronic exercise may reduce intratumoral hypoxia, downregulating HIF-1α and its pro-angiogenic targets such as VEGF, as observed in breast tumor models, where exercise led to lower HIF-1α levels and slower tumor growth [32, 42, 80]. This improved oxygenation promotes vascular normalization, marked by more organized, pericyte-covered vessels, similar to the effects of anti-angiogenic therapies, but through physiological pathways [32]. Acute hemodynamic responses to exercise bouts (e.g., increased shear stress) also stimulate endothelial nitric oxide (NO) production, enhancing transient vasodilation and perfusion. Repeated exercise bouts may thus change the tumor microenvironment, improving vascular function and reducing hypoxia-driven angiogenesis [32, 42, 80]. However, tumor heterogeneity may influence responsiveness, with certain subtypes exhibiting differential reliance on HIF-1α, VEGF, or NO-related pathways. An enhanced understanding of how angiogenic signaling, vasodilatory mechanisms, and shear-stress–driven endothelial activation interact across tumor phenotypes will be essential to determine which tumors derive the greatest benefit from exercise-based vascular modulation [81, 82].

Moving forward, future research should first prioritize conducting well-designed human trials to establish foundational evidence of exercise-induced effects on tumor biology. Once preliminary efficacy and feasibility are demonstrated, efforts can then focus on the standardization of exercise protocols and the rigorous assessment of tumor hemodynamics in clinical settings. A key recommendation is to incorporate advanced imaging techniques—such as contrast-enhanced ultrasound and perfusion measurements—into trial designs to directly monitor changes in tumor blood flow and oxygenation in response to exercise. Establishing a set of validated, sensitive hemodynamic biomarkers will enable researchers to confirm whether a given exercise regimen is “normalizing” the tumor vasculature in patients. Standardization is also needed in how exercise is prescribed and reported. It remains unknown whether a higher intensity or volume of exercise produces proportionally greater improvements in perfusion, or if there is a ceiling effect. Future trials could employ different exercise intensities (e.g., moderate- versus high-intensity training) to determine the minimal and optimal “dose” needed to induce vascular changes without causing undue fatigue or stress in patients. In addition, timing of exercise relative to chemotherapy administration is an open question; some protocols might schedule exercise sessions strategically to maximize drug delivery during the window of increased tumor blood flow. The timing and duration of this potential “window” remain unclear and require further investigation in human models to optimize therapeutic integration. Similar concepts have been discussed in other solid tumors, as shown in reviews by Seet-Lee et al. and Schumacher et al., indicating that exercise-induced vascular modulation may represent a broader mechanism relevant across cancer types. Addressing such questions will likely require multidisciplinary collaborations, integrating oncologists, exercise physiologists, radiologists, and molecular scientists. By using consistent methodologies and investigating these mechanistic details, future studies will be better positioned to clarify how exercise can be harnessed to modulate tumor perfusion. In turn, this knowledge could inform clinical guidelines, potentially leading to exercise recommendations as an adjuvant strategy to improve chemotherapy response in breast cancer. The ultimate goal of this line of research is to enhance patient outcomes by marrying systemic therapy with lifestyle interventions that target the tumor microenvironment—a paradigm shift that transforms empirical observations of exercise benefit into evidence-based clinical practice.

## Conclusions

Taken together, current evidence highlights the pivotal role of tumor blood flow in modulating cancer progression and treatment efficacy. Advanced imaging techniques have improved our ability to precisely assess tumor perfusion. The integration of exercise as a vascular modulator represents a novel and promising therapeutic strategy. Exercise has the potential not only to improve overall health and treatment tolerance but also to normalize tumor vasculature, enhance drug delivery, and reshape the tumor microenvironment. However, to translate these findings into clinical oncology, future research must address methodological gaps, validate reliable perfusion biomarkers, and establish evidence-based exercise protocols tailored to tumor biology. This review reinforces the importance of a multidisciplinary approach to cancer care, in which lifestyle interventions such as exercise can be strategically combined with conventional therapies to improve patient outcomes.

## Declarations

### Data availability statement

No new data were generated or analyzed in support of this review. All data discussed are available in the published literature cited in the manuscript.
